# Crash Prediction Using Deep Learning in a Disorienting Spaceflight Analog Balancing Task

**DOI:** 10.3389/fphys.2022.806357

**Published:** 2022-01-28

**Authors:** Yonglin Wang, Jie Tang, Vivekanand Pandey Vimal, James R. Lackner, Paul DiZio, Pengyu Hong

**Affiliations:** ^1^Computer Science Department, Brandeis University, Waltham, MA, United States; ^2^Ashton Graybiel Spatial Orientation Laboratory, Brandeis University, Waltham, MA, United States; ^3^Volen Center for Complex Systems, Brandeis University, Waltham, MA, United States; ^4^Psychology Department, Brandeis University, Waltham, MA, United States

**Keywords:** spaceflight analog, crash prediction, spatial disorientation (SD), dynamic balance, vehicle control, deep learning—artificial neural network (DL-ANN), vestibular

## Abstract

Were astronauts forced to land on the surface of Mars using manual control of their vehicle, they would not have familiar gravitational cues because Mars’ gravity is only 0.38 g. They could become susceptible to spatial disorientation, potentially causing mission ending crashes. In our earlier studies, we secured blindfolded participants into a Multi-Axis Rotation System (MARS) device that was programmed to behave like an inverted pendulum. Participants used a joystick to stabilize around the balance point. We created a spaceflight analog condition by having participants dynamically balance in the horizontal roll plane, where they did not tilt relative to the gravitational vertical and therefore could not use gravitational cues to determine their position. We found 90% of participants in our spaceflight analog condition reported spatial disorientation and all of them showed it in their data. There was a high rate of crashing into boundaries that were set at ± 60^°^ from the balance point. Our goal was to see whether we could use deep learning to predict the occurrence of crashes before they happened. We used stacked gated recurrent units (GRU) to predict crash events 800 ms in advance with an AUC (area under the curve) value of 99%. When we prioritized reducing false negatives we found it resulted in more false positives. We found that false negatives occurred when participants made destabilizing joystick deflections that rapidly moved the MARS away from the balance point. These unpredictable destabilizing joystick deflections, which occurred in the duration of time after the input data, are likely a result of spatial disorientation. If our model could work in real time, we calculated that immediate human action would result in the prevention of 80.7% of crashes, however, if we accounted for human reaction times (∼400 ms), only 30.3% of crashes could be prevented, suggesting that one solution could be an AI taking temporary control of the spacecraft during these moments.

## Introduction

Spatial disorientation occurs when pilots have an inaccurate perception of their position, motion or attitude and is caused by a variety of factors (Poisson and Miller, 2014). Studies have estimated that 90–100% of pilots have experienced it (Newman, 2007; Gibb et al., 2011). A majority of fatal aircraft accidents caused by spatial disorientation occur when pilots are unaware that they are disoriented (Braithwaite et al., 1998). Astronauts will similarly be susceptible to spatial disorientation during gravitational transitions such as when landing on the surface of a planet or the Moon (Shelhamer, 2015; Clément et al., 2020) because they will not have access to familiar gravitational cues and they will have previously undergone sensorimotor adaptions to weightlessness which are not fully understood. Long duration spaceflight will also add multiple simultaneous stressors, such as radiation, psychological problems (e.g., isolation, anxiety, depression), physiological changes (cardiovascular, bone, muscle, visual, and vestibular systems), which may heighten the effects of spatial disorientation (Clément et al., 2020). Despite improvements in technology, rates of spatial disorientation are not significantly reducing (Daiker et al., 2018). There are very few studies that have attempted to develop an alerting system that can predict crashes when a pilot is disoriented. Daiker et al. (2018) proposed a proof-of-concept idea for NASA’s Cost Effective Devices for Alerting Research (CEDAR), where they used a model of the vestibular system, aircraft dynamics, and sensors to create a predictive alerting model. Though deep learning has been used successfully in predicting the outcome of manual control errors (Zgonnikova et al., 2016), no study has used deep learning methods such as artificial neural networks to predict crashes in situations where participants are spatially disoriented. Our study is in line with the NASA Human Research Program Roadmap revised July 2021 (NASA, 2021) that lists “Sensorimotor Manual Control Countermeasure Development” as a critical task and designates two critical research gaps: (1) “Characterize the effects of short and long-duration weightlessness on manual control (fine motor control) after G transitions.” (SM-102), and (2) “Develop and test manual control countermeasures, such as vibrotactile assistance vest, and other human factors aids” (SM-202). The Roadmap citation describing such countermeasures explicitly mentions the use of AI targeting human-automation task sharing. Attempts to address these gaps under operational spaceflight conditions have been hampered by the limited access to astronauts close to the G transition of landing (Moore et al., 2019), and the Roadmap explicitly calls for studies like ours with relevant partial analogs.

**FIGURE 1:**
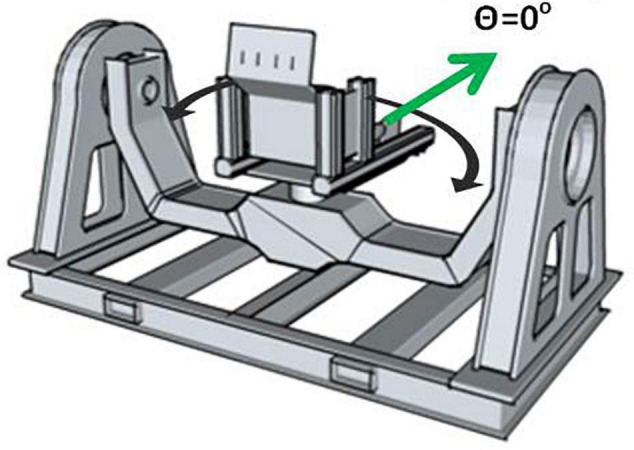
MARS in the horizontal roll plane with the balance point at θ = 0°.

We created a spaceflight analog task that led to disorientation by securing blindfolded participants into our Multi-axis Rotation System Device (MARS) in the horizontal roll plane (Figure 1). In this plane, participants do not tilt relative to the gravitational vertical and as a result cannot use gravity dependent otolith and somatosensory shear forces to obtain a sense of their angular position. To determine their angular position, they can only use motion cues detected by the semicircular canals and rapidly adapting somatosensory receptors. We programmed the MARS with inverted pendulum dynamics because of its relevance to unstable vehicle control. Participants were instructed to use an attached joystick to stabilize themselves around the balance point (θ = 0^°^) (Panic et al., 2015, 2017; Vimal et al., 2016, 2017, 2018, 2019). Crash boundaries were set at ± 60^°^ from the balance point, and when it was reached the MARS would reset back to the balance point. In this condition, 90% of participants reported spatial disorientation and all of them showed it in their data as a pattern of positional drifting. Compared to the control condition (Vertical Roll Plane, where gravitational cues from tilt were available), the rate of crashes was significantly higher and collectively participants in the horizontal roll plane showed poor performance and minimal learning across trials (Vimal et al., 2017, 2019). Our goal was to train and compare recurrent neural networks (RNN) and non-RNN deep learning models to predict the occurrence of crashes before they happened in our disorienting spaceflight analog task.

## Materials and Methods

### Data Collection

To build the deep learning models we used data from Vimal et al. (2017; 2019; 2020).

#### Participants

34 healthy adult participants (18 females and 16 males, 20.4 ± 2.0 years old) gave written consent to participate in the experiments approved by the Brandeis Institutional Review Board. In our prior work (Vimal et al., 2020) we showed that these participants span a large range of learning and performance and could be clustered into three groups: Proficient, Somewhat Proficient and Not Proficient. The Proficient group showed significant learning across trials for majority of our metrics, whereas the Not-Proficient group acquired a suboptimal strategy that led to worsening performance in most metrics over time.

#### Equipment

The MARS was programmed with inverted pendulum dynamics about a horizontal roll axis (Figure 1) using the equation, $\ddot{\theta }={\text{k}}_{\text{P}}\,\text{sin}\,\theta$, where θ is the angular deviation from the direction of balance (DOB) in degrees, and k_*P*_ is the pendulum constant. To make the task challenging, we used a pendulum constant of 600^°^/s^2^ (≈0.52 Hz) based on our prior work (Vimal et al., 2016, 2017, 2018, 2019). “Crash” boundaries were set at ± 60^°^ from the direction of balance. Angular velocity was limited to ± 300^°^/s, and angular acceleration to ± 180^°^/s^2^. At every time step (∼0.02 s), a velocity increment proportional to the joystick deflection was added to the MARS velocity and computed by a Runge-Kutta RK4 solver (Lambert, 1973) to calculate the new MARS angular position and velocity.

#### Procedure

Participants were blindfolded and wore noise canceling headphones that played white noise. They were secured in the MARS with a five-point harness, a lap belt, lateral support plates and foot straps (Figure 1). Their heads were stabilized using a U-shaped frame cushioned with foam that was attached to the MARS. To prevent visual or auditory cues, they were blindfolded and wore earplugs and noise canceling headphones that played white noise. A Logitech Freedom 2.4 cordless joystick was attached to the right arm rest and a “kill switch” that the participant could press to stop the experiment was attached to the left arm rest. No participant ever used the kill switch.

Prior to data collection, participants watched a video of a person balancing the MARS in the horizontal roll plane and of the MARS reaching the “crash boundaries” at ± 60^°^ from the balance point and then resetting. They were told that the MARS behaved like an inverted pendulum and they were instructed to use the joystick to minimize oscillations about the balance point, which was always at 0^°^ (their starting position). The trial started with an auditory “begin” and whenever participants reached the crash boundaries, they heard “lost control, resetting.” As the MARS automatically reset to the start position at a rate of 5^°^/s, the joystick was disabled. Once at the reset position, which was always 0^°^, they heard an auditory “begin” command and the joystick was simultaneously enabled. Participants balanced in two sessions conducted on consecutive days. On each day they underwent five blocks of four trials, with each trial consisting of 100 cumulative seconds of balancing, excluding the reset times after crashes, or a total elapsed time of 150 s. After every four trials participants were brought to an upright orientation and were given a 2 min break during which they were questioned about any symptoms of motion sickness. They were given no verbal feedback about their performance.

### Crash Prediction by Deep Learning

Our goal was to predict the occurrence of crashes before they happened. We used a classification approach where we took windows of data (MARS angular position and velocity, and joystick deflection) and then predicted whether a crash would happen later or not (i.e., classified the data segment as leading to a crash or non-crash). We trained deep learning models, which is a machine learning method that uses models consisting of artificial neural networks. Deep learning models can automatically extract often obscured patterns in intricate, high-dimensional data of large quantity (LeCun et al., 2015), thereby qualifying as excellent choices for our classification task.

#### Preprocessing Data Into Episodes

**FIGURE 2:**
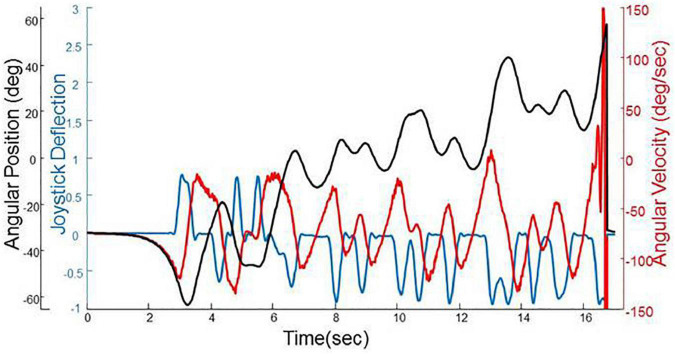
A segment of trial data from a representative participant showing angular position (black), angular velocity (red) and joystick deflection (blue). The characteristic pattern of positional drifting can be seen to end with the participant hitting the crash boundary at 60^°^.

First we extracted all of the segments of data (angular position, velocity, and joystick deflection) that were continuous, under human control, and did not have any crashes. We refer to these segments of data as episodes and Figure 2 provides an example, which shows the characteristic pattern of positional drifting (in black) where a representative participant oscillates away from the balance point at 0° until they hit the crash boundary at 60°. By this definition, there were 21,469 episodes in total. We split all of the episodes so that 90% of them (19,322 in total) were used as the training set to train the deep learning models and the rest (2,147 in total) were used as the test set to evaluate the effectiveness of the models.

**FIGURE 3:**
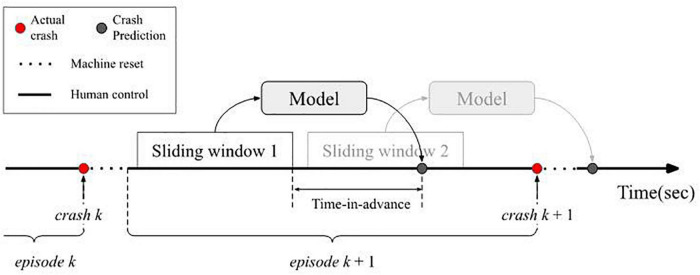
When training the model, sliding windows between crash events sent data to the model along with either a 0 or 1 to indicate whether a crash occurred in the time-in-advance. During testing, the sliding window inputted data to the model and the model output whether a crash would occur or not in the time-in-advance duration.

For every episode, to generate training samples, we slid a fixed size window from a crash event to the next one and extracted the MARS angular position, MARS angular velocity and joystick deflections (Figure 3). This process excluded the windows that overlapped with the MARS’ resetting times (after a crash, the MARS resets to the 0^°^ point and participants have no control). A sample was labeled as a “crash” if a crash happened within the “time-in-advance” interval, e.g., in Figure 3, “sliding window 2” would be labeled as a crash. Otherwise, it was labeled as a “non-crash,” e.g., sliding window 1 in Figure 3. These windows were used as input and the labels as the correct answer, in order to either train the model to predict the correct label or to evaluate whether the trained model predicted the label correctly.

Previous work found, unexpectedly, that a percentage of joystick deflections were made in the same direction as MARS angular position and velocity, accelerating the MARS toward rather than away from the crash boundaries. These are called “destabilizing joystick deflections” and are indicative of non-proficient control patterns (Vimal et al., 2020). Because of their importance, we engineered destabilizing joystick deflections as an additional feature where it was defined as a Boolean feature that was true if position, velocity, and joystick deflection all had the same sign. That is, for any given sliding window, for every time point, we extracted MARS angular position, velocity and joystick deflections, and in addition, for every time point, we had either a 0 (not a destabilizing joystick point) or a 1 (destabilizing joystick point).

To prevent data leakage where portions of the same data were used in both the training and testing phases, we made certain that all sliding window samples from a given episode were either in the training pool or the testing pool, but not both.

#### Deep Learning Algorithms

There exist a wide variety of deep learning models, each suitable for a different range of tasks. For our classification task on time-series data, to enable shorter latency in future real-world implementations where computational resources may be limited, we opted for models with lower complexity and implemented multilayer perceptron (MLP), convolutional neural network (CNN), long short-term memory (LSTM), gated recurrent unit (GRU), stacked LSTM and stacked GRU. All models mentioned in this paper were built with Keras and TensorFlow and used binary cross-entropy as the loss function and sigmoid as the activation function for the final output layer. The descriptions of the hardware and the training procedure can be found in Supplementary Material.

##### Multilayer Perceptron

The MLP is the classical type of deep learning neural network. It usually contains a few feed-forward fully connected layers of neurons (Rosenblatt, 1961).

After manual tuning, the chosen MLP consisted of five layers—an input layer, three hidden layers, and an output layer. The input layer flattens the input data matrix into a vector. The vector is then passed through the hidden layers, which each has 50 neurons and rectified linear unit (ReLU) as its activation function.

##### Convolutional Neural Network

The CNN works well with data that has spatial relationship. It was originally developed for image processing (LeCun et al., 1989) and can also be applied to processing temporal sequences (e.g., Kim, 2014). It uses convolution kernels to slide along input sequences and pooling layers to down-sample kernel output; CNN therefore can provide time-invariant responses, meaning that it can be sensitive to similar input patterns that appear at different time steps.

The structure of the optimized CNN in this experiment was as follows: the first convolution layer (implemented with Conv1D) had 128 filters of size 3 and a stride of 1, with ReLU as its activation function, and padding such that the output had the same length as the input; this convolution layer was then followed by a max pooling layer of size 2. Then, the output of the first max pooling layer was passed to another set of convolution layer and max pooling layer that was the same as the first convolution layer except for having 128 files of size 4. Before the final output layer, the result of the second max pooling layer was then flattened and passed to a feedforward neural network with 100 neurons and ReLU as its activation function.

##### Long Short-Term Memory

The LSTM model is one type of recurrent neural network (RNN) which feeds the outputs, namely cell state and hidden state, of previous time steps back onto itself (Hochreiter and Schmidhuber, 1997). An LSTM module contains three gates (i.e., input gate, output gate, and forget gate) which control the information flow throughout the module and allow it to keep track of longer-term dependencies in the input sequences in contrast to classic recurrent neural networks.

The following describes the configuration of the LSTM model after manual optimization. The LSTM had a hidden layer of size 100, and the final hidden state output of the LSTM model was passed to a dropout layer with a rate of 0.5, followed by a fully connected layer of size 128 with Rectified Linear Unit (ReLU) activations. The final output layer was added after the fully connected layer.

##### Gated Recurrent Unit

The GRU model is another type of RNN, similar to LSTM in terms of using gates to control its internal information flows. Unlike LSTM, GRU does not have a separate cell state and only has a hidden state (Cho et al., 2014). This simplification makes a GRU have fewer parameters than a LSTM, and hence sometimes easier to train.

The following describes the configuration of the GRU model after manual optimization. Similar to the LSTM model in our experiment, the GRU module had a hidden layer of size 100, and its output was passed to a dropout layer with a rate of 0.5, followed by a fully connected layer of size 128 with Rectified Linear Unit (ReLU) activations and the final output layer.

##### Stacked Recurrent Neural Networks

In addition to using a single RNN module in the mode, we also experimented with including additional modules stacked on top of the original one, namely, stacked LSTM and stacked GRU.

A stacked LSTM stacks multiple LSTMs on top of one another (Pascanu et al., 2013; Sha and Hong, 2017; Peters et al., 2018). A LSTM in a top layer takes the hidden state outputs of the LSTM below it and feeds its outputs to another LSTM above it. This arrangement allows the whole model to capture longer and multiple-resolution time-dependencies.

We trained with a double-layer stacked LSTM, which had two LSTM modules stacked as described. Both modules had hidden states of size 100. We then took the hidden state output of the top-layer LSTM and fed it to a fully connected layer of 128 neurons with ReLU activations, followed by the final output layer.

A stacked GRU adopts the same stacked architecture of a stacked LSTM but with two GRU modules (Sun et al., 2020).

The double-layer stacked GRU model trained in our experiment was similar to the stacked LSTM model with both modules having hidden states of size 100. The final hidden state output passed to a 128-neuron, ReLU activated fully connected layer, then eventually passed to the final output layer.

##### Non-deep Baseline Model

In addition to the deep learning algorithms, we also implemented the simple linear classifier (Cox, 1958), which transforms a simple linear combination of the input features to the output by a sigmoid function. This model provides a baseline for our comparisons. We will demonstrate that simple linear models do not perform as well as deep learning models in complex tasks such as our spaceflight analog condition. A significant advantage of using deep learning approaches is that they can be applied to the raw inputs and achieve similar or better performance.

#### Cross Validation

To determine how well our models were performing against each other on the training set and how the best model could perform on an unseen test set, we evaluated the deep learning classifiers with 10-fold cross-validation (CV). In 10-fold CV, the training dataset is divided into 10 non-overlapping subsets. We used each subset to evaluate a model trained on the other nine subsets. Finally, we took the metrics averaged over 10 folds as an estimate of how the model would perform on the entire training set. After we identified the best model with the highest CV metrics, we kept the model from the best performing fold for final evaluation on the test set.

#### Evaluation of Model Performance

We evaluated model performance initially with “Area Under the Receiver Operating Characteristic Curve” (AUC), a threshold invariant metric for model selection during 10-fold CV. In our case, AUC represents the probability that the model ranks a random non-crash example as less likely to crash than a random crash sample, which is the desired behavior. Therefore, a higher AUC would mean that the model is better trained and separates crash samples more clearly from non-crash samples.

While high AUC may reflect how well the model distinguishes the positive and negative labels, it does not provide a full view of how useful the model would be in practice, where cost-driven threshold tuning is crucial. For example, in aviation and spaceflight applications, false negatives will have much higher costs than false positives. Table 1 illustrates the four types of predictions. False negatives, where the model incorrectly predicts that no crash will occur, may lead to death, depending on how the algorithm decision is implemented (warning to pilots vs. pilot override). In contrast, false positives, where the model incorrectly predicts that a crash will occur, will result in the pilot being on alert. Therefore, in our spaceflight analog task, we want to minimize false negatives. There are two measures that allow us to quantify these concepts:

**TABLE 1:** Definition of the four types of prediction.

	Actual crash	Actual non-crash
**Model predicted crash**	True positive	False positive
**Model predicted non-crash**	False negative	True negative

Recall is defined by:


$$\text{Recall}=\frac{\mathrm{True}\,\mathrm{Positive}}{\mathrm{True}\,\mathrm{Positive}+\mathrm{False}\,\mathrm{Negative}}$$

Thus, a higher recall in our application means a higher percentage of all crash samples being correctly classified as crashes by the model, hence fewer missed crashes.

Precision is defined by:


$$\text{Precision}=\frac{\mathrm{True}\,\mathrm{Positive}}{\mathrm{True}\,\mathrm{Positive}+\mathrm{False}\,\mathrm{Positive}}$$

In our application, a higher precision means higher percentage of the model’s crash predictions are correct.

When evaluating the same model, as we move the model’s decision threshold to different values, we would have different pairs of precision and recall values. One common way to evaluate using these threshold sensitive metrics is to set a predefined value on either precision or recall and calculate the corresponding value of the other metric. Based on the cost analysis and definitions above, when evaluating the performance of our models, we prioritized minimizing false negatives and we preferred models with a high recall value, which we set to be 95%. At a recall of 95%, we then looked at which models had greater precision and we refer to this selection criteria as P@0.95R.

## Results

### Model Selection

We applied 10-fold cross-validation, AUC, and precision at recall to evaluate the performance of our approach. In addition to prediction accuracy, we also wanted to predict crashes as early as possible.

Table 2 shows the P@0.95R averaged over 10-fold CV for each window size, time-in-advance duration, and model type combination. We found that the simple linear model performed extremely poorly when compared to the deep learning models when there are only raw machine readings (i.e., angular position and velocity, joystick deflection) and one manually engineered Boolean feature (destabilizing joystick deflections) available without extensive feature engineering. The advantage of using deep learning models is that they can automatically learn representations (i.e., features) from the same raw data with minimal feature engineering and achieve much better results.

**TABLE 2:** Precision at 0.95 recall (P@0.95R) scores averaged over 10-fold cross-validation for each model type, window size, and time-in-advance combinations.

Models	Window size	P@0.95R at time-in-advance (%)		
		300 ms	600 ms	1,000 ms
Simple linear model	500 ms	2.95 ± 0.51	3.28 ± 0.21	5.25 ± 0.33
	1,000 ms	2.72 ± 0.57	3.37 ± 0.15	5.4 ± 0.38
	1,500 ms	2.82 ± 0.6	3.5 ± 0.16	5.43 ± 0.27
MLP	500 ms	78.63 ± 6.01	66.9 ± 2.43	29.01 ± 1.45
	1,000 ms	85.98 ± 5.31	67.14 ± 2.99	30.99 ± 1.17
	1,500 ms	74.23 ± 14.88	65.79 ± 3.71	31.19 ± 1.67
CNN	500 ms	84.01 ± 3.89	65.45 ± 3	28.72 ± 1.71
	1,000 ms	82.25 ± 5.34	65.76 ± 4.86	30.47 ± 1.32
	1,500 ms	85.25 ± 5.96	67.58 ± 3.95	32.28 ± 1.4
LSTM	500 ms	92.02 ± 3.6	75.41 ± 3.91	31.71 ± 1.62
	1,000 ms	90.38 ± 5.99	75.16 ± 3.11	34.34 ± 2.49
	1,500 ms	91.08 ± 5.63	72.94 ± 2.68	34.24 ± 1.8
GRU	500 ms	91.94 ± 5.73	75.05 ± 3.26	32.2 ± 2.27
	1,000 ms	91.44 ± 3.32	74.36 ± 3.23	34.05 ± 2.17
	1,500 ms	90.62 ± 5.89	71.35 ± 2.94	34.35 ± 1.61
Stacked LSTM	500 ms	89.8 ± 7.85	75.21 ± 2.48	32.59 ± 1.72
	1,000 ms	93.34 ± 3.25	76.18 ± 3.99	34.74 ± 2.36
	1,500 ms	92.46 ± 3.76	76.88 ± 1.32	34.93 ± 1.64
Stacked GRU	500 ms	89.7 ± 6.58	76.63 ± 2.63	32.83 ± 1.45
	1,000 ms	92.02 ± 4.98	76.47 ± 2.62	34.77 ± 1.84
	1,500 ms	90.42 ± 6.73	74.92 ± 1.74	35.81 ± 1.87

**FIGURE 4:**
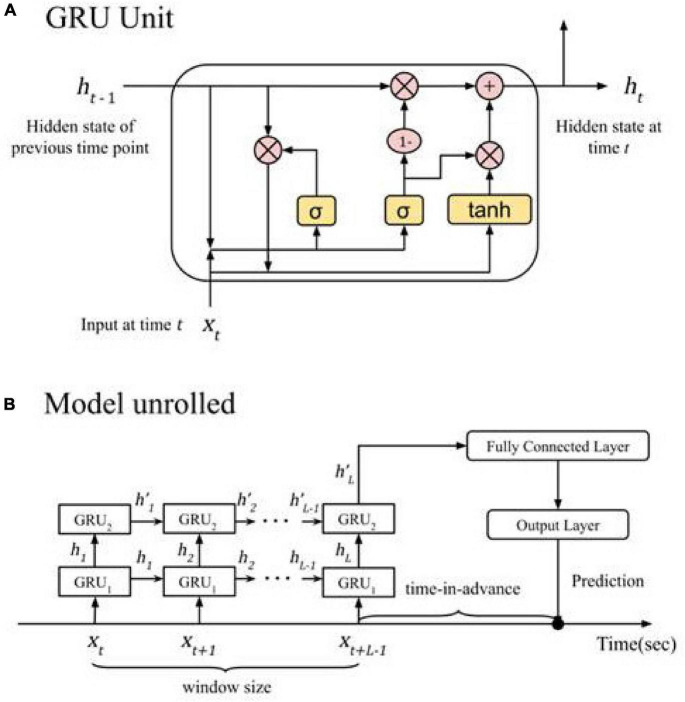
The final crash prediction model. **(A)** A close-up of the GRU cell; pink circles represent element-wise matrix operations, yellow blocks represent the activation functions. It is recurrent in that the hidden state of the previous time step (h_*t*–1_) is fed back to the same GRU unit to generate the new hidden state at the current time step (h_*t*_) **(B)** the two GRU modules in the stacked GRU model unroll to process the data at each time step in the input window and produce a prediction of whether a crash will happen. At time t, GRU_1_ takes feature values (x_*t*_) as input, while GRU_2_ takes the hidden state output as its input. After the last time step, the hidden state output of GRU_2_ (h’_*L*_) is passed to a feedforward neural network to generate a prediction.

Among the deep learning models in Table 2, we found lower precision values for the set value of 95% recall for non-RNN such as MLP and CNN. We determined stacked GRU to be the best based on the following reasons. Overall, stacked-RNNs performed somewhat better than single-layer RNNs in terms of CV results. Furthermore, we chose stacked GRU for detailed examination because the time to train a stacked GRU (average 12.63 h per 10-fold CV) is much shorter than stacked LSTM (average 15.01 h per 10-fold CV), without notable degradation in evaluation metrics. Therefore we chose stacked GRU because it performed better than single layer RNNs and performed at the same level as LSTM while taking less computational time. Figure 4 shows the detailed architecture of the chosen stacked-GRU model.

### Time-in-Advance Prediction

We chose a data window size of 1,000 ms to capture enough joystick behavior because participants on average make full joystick deflections at 1–2 Hz (Vimal et al., 2016, 2017, 2018). Additionally, we did not find large changes in model performance with different data window sizes (see Supplementary Figure 1).

**FIGURE 5:**
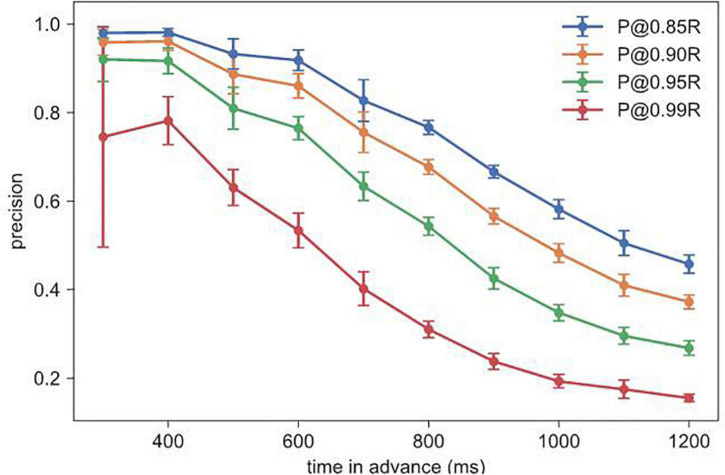
Precision at different recall values and time-in-advance durations, for stacked GRU at 1,000 ms window size.

We found a tradeoff between recall and precision in all model types, where setting a higher recall empirically resulted in a lower precision (e.g., stacked GRU as shown in Figure 5). Greater time-in-advance durations resulted in lower values of precision (Figure 5).

We wanted time-in-advance duration to be as long as possible, so that crash prediction can be generated as soon as possible. As a summary number for prediction capability, we chose 800 ms as the best time-in-advance duration which resulted in a 10-fold CV AUC of 0.9927 ± 0.0006 and P@0.95R of 0.5432 ± 0.0203. The model takes on average 30 ms to classify one data window on a 2.5 GHz Intel Xeon CPU, which should impose few latency issues on real-time implementation.

### Analysis of Results at High Recall

**FIGURE 6:**
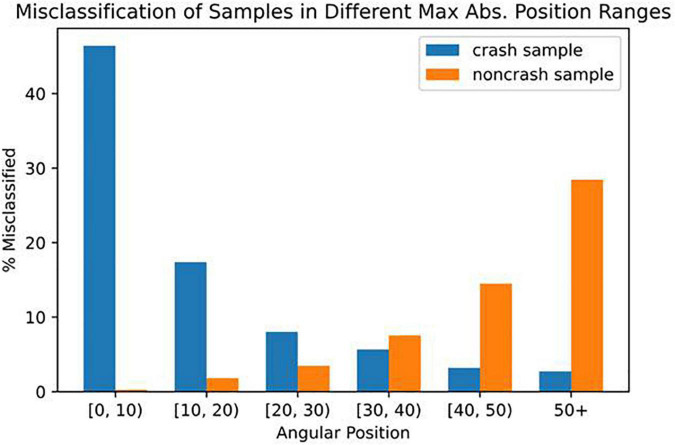
Percentage of misclassified crash samples (blue) or non-crash samples (orange) out of all samples within a range of the largest magnitude of angular positions in the data window.

To further understand our model’s capabilities, we examined those crashes that were misclassified as non-crash (i.e., false negatives) by the model even at a recall as high as 95%. In Figure 6, we plotted the rate of the model misclassifying crash samples as non-crash (blue) and non-crash samples as crashes (orange), grouped by the farthest the participant had been from the balance point in the data window, i.e., the largest magnitude of angular position. We found that the crashes which were the hardest for the model to correctly identify were those that occurred when participants were near the balance point (0^°^) where over 46% of crash samples were mislabeled as non-crashes.

To understand what caused a crash at such a seemingly safe location of the 0^°^ point, we examined the data in the 800 ms time-in-advance duration following these false negative inputs, i.e., outside of the 1,000 ms sliding window data available to the model. We found that participants were making destabilizing joystick deflections in the time-in-advance duration that led to the crashes. Table 3 shows that the percentage of destabilizing joystick deflections in the time-in-advance duration was the greatest for false negatives (Table 3), meaning that participants often made unexpected destabilizing joystick deflections in the time-in-advance portion which was unavailable to the model. This suggests that the reason the model predicted “no crash” was because the participants were performing well near the balance point but then unexpectedly initiated a destabilizing joystick deflection because they were disoriented and did not have a clear sense of their orientation. True positives had the smallest percentage because critical errors had already been made which would lead to a crash.

**TABLE 3:** Percentage of predictions containing unexpected destabilizing joystick deflection in time-in-advance duration.

Type of prediction	False negative	False positive	True negative	True positive
**% of DJD**	67.50%	58.92%	53.94%	33.64%

**FIGURE 7:**
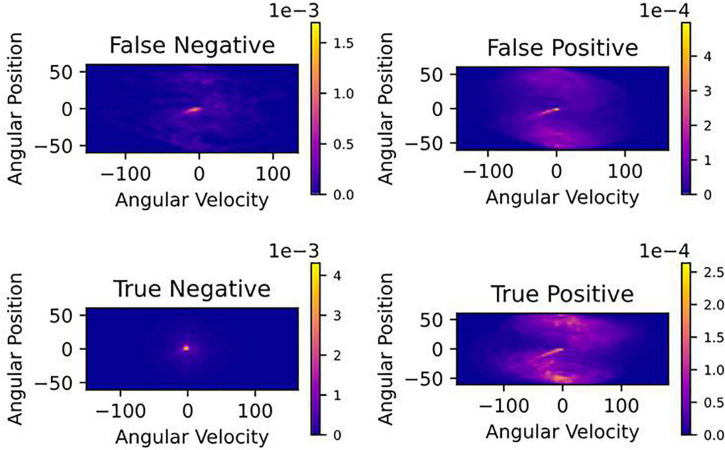
Density maps of the velocity and position at all-time steps in time-in-advance duration.

Figure 6 also revealed that false positives (model incorrectly predicted a crash would happen) were more likely to occur at large angular positions near the crash boundaries (±60^°^). Because we prioritized reducing false negatives (having a high recall), we had a higher rate of false positives (low precision). To further understand when false positives occurred, in Figure 7, we created a density map of angular position and velocity. We found that false positives had higher angular position and velocity, suggesting that the model was identifying dangerous behavior as being potential places of crashes. Similar results can be seen for true positives (model correctly predicts a crash will occur) because the greatest danger was at high values of position and velocity.

## Discussion

Our objective was to create a model that could predict the occurrence of crashes in a stabilization task where participants were spatially disoriented similar to what astronauts may experience. We used the angular position, velocity and joystick deflections from the stabilization task to train a stacked GRU model to predict whether a crash would occur at a certain future time point. We chose stacked GRU over other models because its recurrent neural network (RNN) structure gave it an advantage for analyzing our time sequence data over non-RNN methods. Within the RNN methods, stacked GRU performed slightly more efficiently (Table 2). Based on the obtained AUC (Area Under the Receiver Operating Characteristic Curve), we found that our model performs well at predicting crashes as early as 1,500 ms before (Supplementary Figure 1). However, for aviation and spaceflight applications, false negative errors (model incorrectly predicts no crash) would be considered much worse than false positive errors (model incorrectly predicts that a crash will occur). For this reason we prioritized minimizing false negatives by setting the recall very high (95%), which resulted in a higher rate of false positives (i.e., lower precision, Figure 5). Please refer to the equations in section “Evaluation of Model Performance” for the definitions of precision and recall.

To understand and characterize the types of crashes the model could not predict even at a high recall value, we plotted the rate of the model misclassifying crash samples as being non-crashes (Figure 6). We were surprised to find that many of the false negative crashes started near the equilibrium point (0°) which many would consider the safest place to be. We discovered that the model’s inability to predict this group of false negative crashes was because participants made unexpected destabilizing joystick deflections in the time-in-advance duration (which the model did not have access to) that caused the device to rapidly accelerate away from the balance point (Table 3). These destabilizing joystick deflections were likely made because participants in the horizontal roll plane are often disoriented and have inaccurate perception of their angular position (Vimal et al., 2021).

Because we chose to set a high recall (minimizing false negatives) we had a high rate of false positives (Figure 5). We were curious about when the model classified false positives. Figure 6 reveals that false positives were occurring at large values of angular position near the crash boundaries (± 60^°^). In Figure 7, we found that false positives occurred at very large magnitudes of angular position and velocity. For this reason, it is possible that false positives could serve as a warning signal for pilots who may be disoriented and reach dangerous angular deviations and velocities. Having too high a false positive rate could result in pilots losing trust with the model. It is not well understood what values of false positives will maintain trust between pilots and Artificial Intelligence (AI), or what level of trust in the AI is useful because case studies show that both too much and too little trust in the technology can lead to fatal accidents in ships and aircraft (Wickens, 1995; Dalcher, 2007; Hoff and Bashir, 2015). Our future work will explore the role of trust and the rate of acceptable false positives.

**FIGURE 8:**
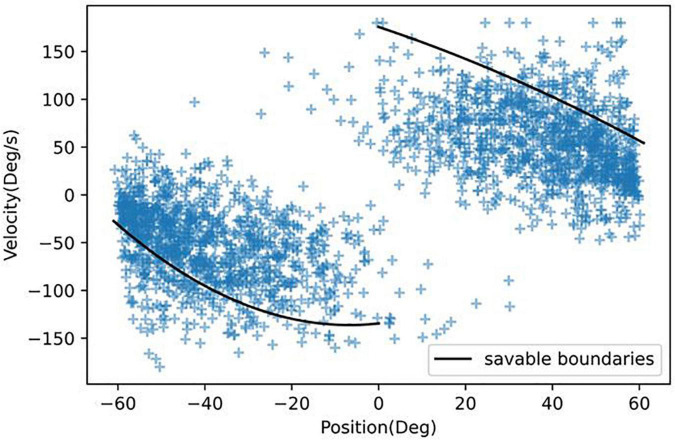
Angular position and velocity 800 ms before all crashes. Those points inside the boundaries can avoid a crash.

Figure 5 shows that the model can predict a crash 800 ms in advance at a high recall (0.95 recall and 0.50 precision). However, would a warning signal at this point prevent any crashes? In Figure 8 we plotted the angular position and velocity 800 ms before all crashes. In our previous work, we had measured boundaries, which if reached, would result in unavoidable crashes (Vimal et al., 2016, 2017). All points in Figure 8 that are inside the boundaries are therefore considered recoverable.

In Table 4, we found that if immediate control were taken, 80.7% of crashes could be avoided. Humans can respond to stimuli in 250 ms (Barnett-Cowan and Harris, 2009); however, to respond to more complex cues that require joystick deflections, participants would likely need 400 ms (Berryhill et al., 2005). Table 4 shows that with increasing reaction times, receiving a warning would not be sufficient to prevent crashes. Therefore because of the low precision value and short reaction time, our model would not be deployable in a purely human controlled situation. However, this could be resolved if the AI system took temporary control of the spacecraft or aircraft. For example, Figure 5 shows that with a 400 ms time-in-advance duration, we can obtain a recall value of 95% and a precision value of 92%. Additionally, as mentioned above, many false positives occur at large values of angular position and velocity (Figure 7). In future work, in addition to the deep learning crash detection model, we will also create a warning system to detect dangerous conditions such as large values of angular position and velocity, which will further reduce the false positives and therefore increase the precision.

**TABLE 4:** Percentage of avoidable crashes reduces as prediction time elapses.

Time after window ends	0 ms	200 ms	400 ms	600 ms	800 ms
**% savable**	80.71%	55.42%	30.30%	8.54%	0%

Our current model is trained on data related to MARS “crashes.” However when away from Earth, in a spaceflight condition, astronauts will not be able to safely acquire significant data on crashes. In future studies, we will train the model to identify different levels of danger (as opposed to only crashes), such as poor patterns of behavior (e.g., destabilizing joystick deflections) and dangerous situations (e.g., high angular positions and velocities). In practice, this would mean that as astronauts acquire new data, which can include suboptimal performance, our methodology could be used to train the model and set parameters that identify poor performance or they could label it themselves. In the future, we aim to communicate the warnings generated from the deep learning model as well as body orientation through vibrotactile feedback which has been previously shown to be useful for preventing spatial disorientation during air flight (Rupert, 2000).

## Conclusion

Some studies estimate 90–100% of pilots have experienced spatial disorientation and it is a leading cause of fatal aircraft accidents (Newman, 2007; Gibb et al., 2011). In the present study, we used data from a spaceflight analog balancing task that reliably led to spatial disorientation and loss of control. The deep learning and AI communities have explored problems related to crash avoidance for conventional ground vehicles (Peng et al., 2019), autonomous vehicles (Perumal et al., 2021), unmanned aerial vehicles (Gandhi et al., 2017), ships (Perera, 2018), swarming systems (Lan et al., 2020), and aircraft collisions with other aircraft (Julian et al., 2019). However, no one to our knowledge has used deep learning to predict the occurrence of crashes in a novel analog condition where participants experience disorientation similar to what pilots and astronauts may experience.

Space exploration will often demand astronauts to solve problems independently because of factors such as time delays in communication with Earth (Cooke and Hine, 2002). Additionally, because astronauts will be exploring novel environments, optimal solutions to problems will not be known. These problems can arise both in the short term, such as spatial disorientation experienced during gravitational transitions and in the longer term such as effects to the brain from sustained long duration spaceflight (Roberts et al., 2017). The consequences this will have on spaceflight are not fully understood especially when combined with multiple simultaneous stressors caused by factors such as radiation, psychological and physiological changes (Clément et al., 2020). Therefore, it is important to develop countermeasures such as artificial intelligence systems that are tested under a range of spaceflight analog conditions on Earth and can learn and adapt as astronauts collect data in space.

In our approach we develop an artificial intelligence system that does not have prior knowledge of the paradigm (such as inverted pendulum dynamics and the human vestibular system) and is not trained on optimal behavior (such as in the vertical roll plane where participants have task relevant gravitational cues). Instead our model is trained on data obtained from our disorienting spaceflight analog task. Because our model did not rely on detailed knowledge of the paradigm it suggests that this methodology could be applied to other novel and disorienting conditions such as drifting during brownout (Gaydos et al., 2012), loss of control in human postural balancing in artificial gravity (Bakshi et al., 2020) or from vestibular deficits (Lawson et al., 2016).

## Data Availability Statement

Publicly available datasets were analyzed in this study. This data can be found here: https://figshare.com/s/7d935199c01c0edcafa1.

## Ethics Statement

The studies involving human participants were reviewed and approved by the Brandeis Institutional Review Board. The patients/participants provided their written informed consent to participate in this study.

## Author Contributions

VV designed and ran the spaceflight analog condition and JL and PD advised. PH advised YW and JT who developed the deep learning model and ran all of the simulations. VV, YW, and JT wrote the manuscript. All authors contributed to manuscript revision, read, and approved the submitted version.

## Conflict of Interest

The authors declare that the research was conducted in the absence of any commercial or financial relationships that could be construed as a potential conflict of interest.

## Publisher’s Note

All claims expressed in this article are solely those of the authors and do not necessarily represent those of their affiliated organizations, or those of the publisher, the editors and the reviewers. Any product that may be evaluated in this article, or claim that may be made by its manufacturer, is not guaranteed or endorsed by the publisher.
