# Supplementary material for: Crash Prediction Using Deep Learning in a Disorienting Spaceflight Analog Balancing Task
Source: Front Physiol. 2022 Jan 28;13:806357. doi: 10.3389/fphys.2022.806357 (PMC8832067; doi:10.3389/fphys.2022.806357)
Supplement: Supplementary file 1 [file Data_Sheet_1.docx]

Supplementary Material

# Supplementary Material

## Dataset Imbalance

In our datasets, “non-crash” samples severely outnumbered “crash” samples leading to an imbalanced dataset. Supplementary Table 1 shows that the class imbalance exists for all window sizes and time-in-advance configurations. As time-in-advance shortens, the imbalance ratio increases.

| **window (ms)** | **Time-in-advance (ms)** | **train 0:1 ratio** | **test 0:1 ratio** | **training set total** | **test set total** |
| --- | --- | --- | --- | --- | --- |
| 100 | 300 | 64.005 | 65.991 | 514513 | 59488 |
|  | 600 | 30.396 | 31.790 | 513546 | 60005 |
|  | 800 | 22.269 | 24.897 | 509958 | 63137 |
|  | 1000 | 17.676 | 18.626 | 512723 | 60037 |
|  | 1500 | 11.627 | 10.788 | 518189 | 53752 |
| 500 | 300 | 62.483 | 62.216 | 502467 | 56136 |
|  | 600 | 29.649 | 31.297 | 499699 | 58587 |
|  | 800 | 22.067 | 21.819 | 502782 | 55336 |
|  | 1000 | 17.433 | 17.352 | 502319 | 55514 |
|  | 1500 | 11.408 | 11.217 | 501853 | 55048 |
| 1000 | 300 | 60.930 | 60.104 | 486464 | 53527 |
|  | 600 | 29.351 | 27.046 | 489251 | 50538 |
|  | 800 | 21.580 | 21.301 | 486079 | 53522 |
|  | 1000 | 17.117 | 16.899 | 485999 | 53267 |
|  | 1500 | 11.383 | 10.486 | 487477 | 50424 |
| 1500 | 300 | 59.805 | 57.319 | 470266 | 51787 |
|  | 600 | 28.597 | 28.584 | 469557 | 52245 |
|  | 800 | 21.354 | 20.463 | 471296 | 50202 |
|  | 1000 | 17.080 | 15.790 | 472309 | 48724 |
|  | 1500 | 11.340 | 11.415 | 467090 | 52129 |
| 2000 | 300 | 59.031 | 56.361 | 455634 | 48470 |
|  | 600 | 28.533 | 27.736 | 454249 | 49484 |
|  | 800 | 21.376 | 20.606 | 454501 | 48764 |
|  | 1000 | 17.029 | 17.080 | 452248 | 50497 |
|  | 1500 | 11.569 | 11.243 | 451596 | 48997 |

**Supplementary Table 1:** Experimental settings and corresponding dataset statistics. 0: non-crash, 1: crash.

**Supplementary Figure 1.** Effect of class weights on precision at 0.95 recall (P@0.95R), F1, precision, and recall. Metrics are averaged over 10-fold CV, from stacked GRU model and 1000ms window size, with respect to varying time-in-advance durations. F1, precision, and recall values are calculated by using the default decision threshold of 0.5, while P@0.95R values are calculated by using the lowest decision threshold that yields 0.95 recall. The legends show the class weight ratios of non-crash to crash samples; ‘balanced’ refers to assigning weights to the classes such that the total weight ($\text{class weight × sample size}$) of each class is the same.

To address the class imbalance issue, we experimented with various class weight ratios when designing the loss function, which did not bring significant improvement to our main metric, precision at 0.95 recall, as shown in Supplementary Figure 1. When holding the non-crash class weight constant at 1, we found worse precision at 0.95 recall for crash weight constants that were greater than 1, especially when the time-in-advance durations were shorter.

To our surprise, increasing the weight of the minority (i.e., crash) class did not improve the P@0.95 recall values but instead tended to lower the P@0.95 recall values. To further investigate the issue, we also plotted the F1, precision, and recall values at the default decision threshold used during training, which was 0.5. In Supplementary Figure 1(b), we found the same, if not more drastic, trends in F1 values, where increasing the weight of the crash class resulted in much lower F1 scores. To understand why F1deteriorates, we examine the effects of class reweighting on precision (Supplementary Figure 1(c)) and recall (Supplementary Figure 1(d)) as F1 is the harmonic mean of precision and recall. The plots reveal that, while recall indeed improved when the crash class weight was increased, precision dropped faster than the improvement of recall, which resulted in lower F1 scores.

One possible explanation is that, in our application, re-weighting classes is not only less effective than but also redundant to early stopping during model training. At the beginning of our study, without using early stopping, we found that rebalancing class weights indeed improved the CV results (i.e., AUC). For example, the AUC of LSTM reached around 0.85 in the crash prediction setting of 1000ms window size and 900ms time-in-advance. However, we later found that early stopping alone boosted the AUC of LSTM to over 0.97 in the same crash prediction setting. In addition, as shown above, adding class reweighting to early stopping did not offer any improvement.

## Model Configuration and Training Details

The model training converged on AUC with a patience of 3 epochs. In other words, the training stops after 3 consecutive epochs with the same AUC values. The training converged after an average of 13 epochs over all CV splits in all experiments.

All hyperparameters were manually tuned. All models were implemented using Keras and trained on two NVIDIA RTX 2080Ti GPU cards. For all models, we used the binary cross-entropy loss in the objective function. The Adam optimizer (Kingma and Ba, 2014) was used to train the model with the settings of learning rate = 0.001, beta1 = 0.9, beta2 = 0.999, epsilon = 1e-08, and mini-batch size = 256. The model takes on average 30ms to classify one data window on a 2.5GHz Intel Xeon CPU, which should impose few latency issues on real-time implementation.

We also tried converging on the other main metric, P@0.95R, which consumed more training time (averaging 40 hours per 10-fold CV for stacked GRU) and did not yield any improvement.

# AUC Values

**Supplementary Figure 2.** AUC values from stacked GRU at different time-in-advance duration and window size combinations. We were able to predict the occurrence of crashes as early as 1000ms in advance with high AUC where the window size had minimal effect. However, for the longer time-in-advance duration of 1500ms, the AUC values were much lower and at that point having larger window sizes helped.
